# Analysis of lung cancer-related genetic changes in long-term and low-dose polyhexamethylene guanidine phosphate (PHMG-p) treated human pulmonary alveolar epithelial cells

**DOI:** 10.1186/s40360-022-00559-5

**Published:** 2022-03-30

**Authors:** Hong Lee, Sang Hoon Jeong, Hyejin Lee, Cherry Kim, Yoon Jeong Nam, Ja Young Kang, Myeong Ok Song, Jin Young Choi, Jaeyoung Kim, Eun-Kee Park, Yong-Wook Baek, Ju-Han Lee

**Affiliations:** 1 Medical Science Research Center, Ansan Hospital, Korea University College of Medicine, Ansan-si, Gyeonggi, Republic of Korea; 2 Department of Radiology, Ansan Hospital, Korea University College of Medicine, Ansan-si, Gyeonggi, Republic of Korea; 3 Department of Medical Humanities and Social Medicine, College of Medicine, Kosin University, Busan, Republic of Korea; 4 Environmental Health Research Department, Humidifier Disinfectant Health Center, National Institute of Environmental Research, Incheon, Republic of Korea; 5 Department of Pathology, Ansan Hospital, Korea University College of Medicine, Ansan-si, Gyeonggi, Republic of Korea

**Keywords:** Polyhexamethylene guanidine phosphate, Humidifier disinfectant, Human pulmonary alveolar epithelial cells, Total RNA sequencing, Lung cancer related genes

## Abstract

**Background:**

Lung injury elicited by respiratory exposure to humidifier disinfectants (HDs) is known as HD-associated lung injury (HDLI). Current elucidation of the molecular mechanisms related to HDLI is mostly restricted to fibrotic and inflammatory lung diseases. In our previous report, we found that lung tumors were caused by intratracheal instillation of polyhexamethylene guanidine phosphate (PHMG-p) in a rat model. However, the lung cancer-related genetic changes concomitant with the development of these lung tumors have not yet been fully defined. We aimed to discover the effect of long-term exposure of PHMG-p on normal human lung alveolar cells.

**Methods:**

We investigated whether PHMG-p could increase distorted homeostasis of oncogenes and tumor-suppressor genes, with long-term and low-dose treatment, in human pulmonary alveolar epithelial cells (HPAEpiCs). Total RNA sequencing was performed with cells continuously treated with PHMG-p and harvested after 35 days.

**Results:**

After PHMG-p treatment, genes with transcriptional expression changes of more than 2.0-fold or less than 0.5-fold were identified. Within 10 days of exposure, 2 protein-coding and 5 non-coding genes were selected, whereas in the group treated for 27–35 days, 24 protein-coding and 5 non-coding genes were identified. Furthermore, in the long-term treatment group, 11 of the 15 upregulated genes and 9 of the 14 downregulated genes were reported as oncogenes and tumor suppressor genes in lung cancer, respectively. We also found that 10 genes of the selected 24 protein-coding genes were clinically significant in lung adenocarcinoma patients.

**Conclusions:**

Our findings demonstrate that long-term exposure of human pulmonary normal alveolar cells to low-dose PHMG-p caused genetic changes, mainly in lung cancer-associated genes, in a time-dependent manner.

**Supplementary Information:**

The online version contains supplementary material available at 10.1186/s40360-022-00559-5.

## Background

In Korea, humidifier disinfectants (HDs) have become a national concern. Epidemiological investigations and medical and biological research have revealed that polyhexamethylene guanidine phosphate (PHMG-p), a main constituent of HD, is highly correlated with inflammatory lung fibrosis. These concomitant lung injuries following respiratory exposure to HDs are known as HD-associated lung injuries (HDLI) [1–3].

Although there are many reports on the association between PHMG-p and clinical manifestations, including lung fibrosis and inflammation, there are few long-term and low-dose studies on its carcinogenic potential. There is convincing evidence that studies are needed to elucidate the relationship between PHMG-p and carcinogenesis. First, in our previous studies, the possibility of tumorigenesis in PHMG-p-instilled rat lung was confirmed using computed tomography (CT) image analysis and an elevated expression of several cancer-related genes [4, 5]. Indeed, in our recent 52-week follow-up study on PHMG-p toxicity, we suggested the possibility of PHMG-p as a lung carcinogen by confirming that PHMG-p causes squamous cell carcinoma in the rat lung [6]. Second, although no specific pattern was observed in the relationship between PHMG and malignant neoplasms, it has been reported that the difference in the effect marginally appears in some infants, within the malignant neoplasms of the digestive tract, respiratory and intrathoracic organs, and leukemia [7]. Third, it is reported that polyhexamethylene biguanide (PHMB), which is structurally similar to PHMG, develops angiosarcoma in the liver during oral exposure [8].

Although the cancer-associated pathways affected by PHMG-p treatment have already been elucidated in a human alveolar A549 cell line, which originates from lung carcinoma, the fact that the research focus was on the epithelial to mesenchymal transition (EMT) and that the in vitro research was conducted in lung adenocarcinoma cells, not by normal cells, might be considered limitations in terms of carcinogenesis research [9, 10]. To overcome these limitations, we introduced human pulmonary alveolar epithelial cells (HPAEpiCs) consisting of type I and type II alveolar cells that occupy more than 99% of the internal surface area of ​​the lung. If the cytotoxicity of PHMG-p itself causes acute cell death, long-term subculture cannot be conducted. Therefore, the cytotoxicity of each concentration was measured over a wide range of concentrations and terms, and a concentration that did not cause superficial damage to cells was set and applied to long-term HPAEpiCs sub-culture.

In this study, we showed that PHMG-p induced changes in the transcriptional expression of oncogenes and tumor suppressor genes in terms of lung carcinogenesis in human pulmonary alveolar normal cells through the analysis of total RNA sequencing data. In addition, we designed this study to secure the reliability of candidate genes by comparing three sets of the PHMG-p short-term treatment group and three sets of the long-term treatment group, either individually or together.

## Methods

### Reagents.

Polyhexamethylene guanidine phosphate (PHMG-p) was purchased from BOC Sciences (NY, USA) with CAS registry number 89697–78-9. Cell Counting kit-8 (CCK-8) reagent was obtained from Dojindo (Kumamoto, Japan). TRIzol™ reagent (#15,596,026) and DEPC-treated water (AM9906) were purchased from Thermo Fisher Scientific (MA, USA). Chloroform (C2432-25ML), ethyl alcohol (E7023-1L) and 2-Propanol (278,475-250ML) were obtained from Sigma-Aldrich (MO, USA). TaqMan microRNA reverse transcription kit (#4,366,596) and TaqMan™ universal PCR master mix (#4,304,437) were purchased from Applied Biosystems (MA, USA).

### Cell culture and treatment.

Human pulmonary alveolar epithelial cells (HPAEpiC, #3200; ScienCell Research Laboratories Inc., CA, USA) were maintained in alveolar epithelial cell medium (AEpiCM, #3201; ScienCell) supplemented with epithelial cell growth supplement (EpiCGS, #4152; ScienCell) and 2% (v/v) fetal bovine serum (FBS, #0010; ScienCell), and 1% (v/v) antibiotic solution (P/S, #0503; ScienCell) at 37 °C under saturated humidity in 5% CO_2_. The PHMG-p stock solution was diluted in the culture medium and used at a final concentration of 1 ug/ml, and sub-cultured and maintained in the set condition in which PHMG-p was present for 35 days.

### Cell viability assessment.

HPAEpiCs were incubated in 96-well plates overnight at approximately 80% confluence and treated with PHMG-p (0.25, 0.5, 1, 2, 3, 4, 5, 6, 7, and 8 µg/mL) for 24, 48, and 72 h. CCK-8 reagent (Dojindo, Japan) was used to evaluate cytotoxicity under PHMG-p-containing conditions according to the manufacturer’s instructions. The absorbance of the processed solution containing cells was measured at 450 nm using a microplate reader (SpectraMax M2e; Bucher Biotec, Basel, Switzerland).

### RNA isolation.

Total RNA was isolated using TRIzol reagent according to the manufacturer’s instructions. RNA quality assessment was performed using an Agilent 2100 bioanalyzer using the RNA 6000 Nano Chip (Agilent Technologies, Amstelveen, Netherlands), and RNA samples were quantified using an ND-2000 spectrophotometer (Thermo Inc. DE, USA).

### RT-qPCR analysis.

Isolated RNA was used as a template to synthesize cDNA using the amfiRivert cDNA Synthesis Platinum Master Mix (GenDEPOT, TX, USA). To quantify mRNA levels, real-time quantitative polymerase chain reaction (RT-qPCR) was performed using Power SYBR® Green PCR Master Mix from Applied Biosystems. To assess the levels of *IFI6*, *MX1*, *MMP1*, *ISG15*, *HMGA2*, *PLAT*, *KRT19*, *IL-33*, *TRPA1*, *AK5*, *NT5E*, *PLAU*, and *CDKN1A* mRNA, RT-qPCR was performed using the primers listed in Table S1. The *NDUFA4L2*, *SLITRK6*, *TIMP3*, *COL14A1*, *TBX4*, *FMO3*, *FMO2*, *GPX3*, *HBG1*, *MGP*, and *HBG2* mRNA primers (Table S1) were used to assess the downregulated genes in PHMG-p long-term treated samples. *GAPDH* mRNA was used as the loading control.

### Library preparation and sequencing.

Libraries were prepared from total RNA using the NEBNext® Ultra™ II Directional RNA-Seq Kit (NEW ENGLAND BioLabs Inc., MA, USA). Ribosomal RNA was eliminated using the RiboCop rRNA depletion kit from LEXOGEN Inc. (Vienna, Austria). RNAs that do not contain rRNA were used for cDNA synthesis and shearing, following the manufacturer’s instructions. Indexing was carried out using Illumina indexes 1–12. The enrichment step was performed by PCR. Subsequently, libraries were verified using the Agilent 2100 Bioanalyzer (DNA High Sensitivity Kit) to assess the average fragment size. Quantification was performed using a library quantification kit by applying a StepOne Real-Time PCR System (Life Technologies Inc., CA, USA). High-throughput sequencing was performed as paired-end 100 sequencing using a NovaSeq 6000 (Illumina Inc., CA, USA).

### Data analysis.

Quality control of the raw sequencing data was performed using FastQC [11]. Low-quality reads (< Q20) and adapters were eliminated using FASTX_Trimmer [12] and BBMap [13]. Then, the trimmed reads were mapped to the reference genome using TopHat [14]. Gene expression levels were estimated using fragments per kilobase per million reads (FPKM) by Cufflinks [15]. The FPKM values were normalized based on the quantile normalization method using EdgeR within R [16]. Data mining of mRNA expression profiling and graphic visualization, including Venn diagrams, was carried out using ExDEGA from Ebiogen Inc. (Seoul, Korea). Gene ontology analysis was performed using the Database for Annotation, Visualization, and Integrated Discovery (DAVID) bioinformatics resources using input and output data files obtained from ExDEGA [17, 18]. A hierarchical clustering (HCL) map was created using MultiExperiment Viewer (MeV).

### Kaplan–Meier plot analysis.

To determine the prognostic values of the *ISG15*, *MMP1*, *TRPA1*, *KRT19*, *PLAU, FMO3, COL14A1, FMO2, TIMP3, and SLITRK6* mRNA, transcription levels were measured using a Kaplan–Meier (KM) plotter, an open-source database (www.kmplot.com) that consists of gene expression profiles and survival lifetime data of patients with lung adenocarcinoma. The analysis was performed on a total of 719 lung adenocarcinoma patients. Only the JetSet best probe set was used for analysis. The patients were divided into low (black line) and high (red line) expression levels using the optimal expression cutoff point based on the log-rank test and median split procedure. Other statistical results, including hazard ratios (HRs), 95% confidence intervals, and log-rank P values, were also calculated and presented using this database. Statistical significance was set at *P* < 0.05.

### Statistical analyses.

All data were analyzed using GraphPad Prism v.5.0 (GraphPad Software, CA, USA) and are expressed as the mean ± standard deviation. Statistical significance was set at *P* < 0.05. The log-rank test was used to evaluate the survival differences.

## Results

### PHMG-p-induced cytotoxicity in HPAEpiCs.

**Fig. 1:**
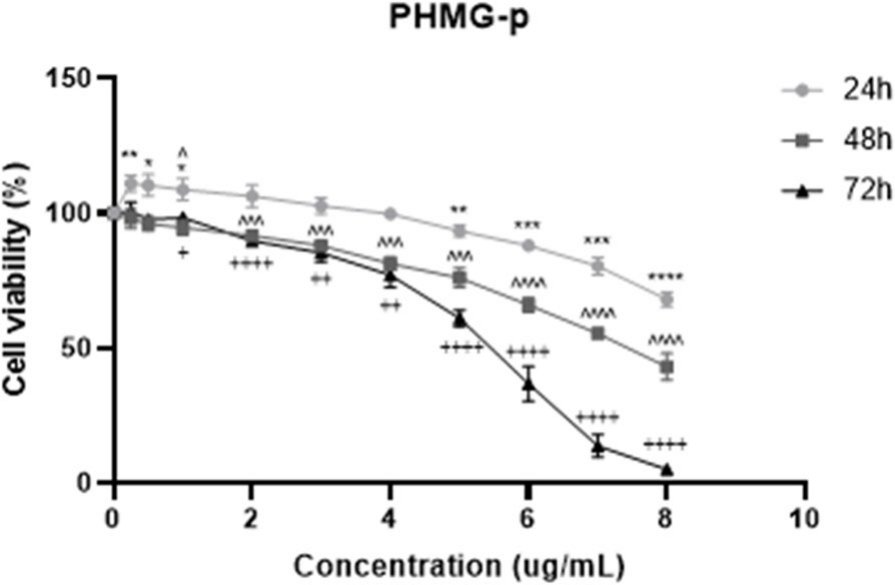
Cell viability evaluation of human pulmonary alveolar epithelial cells (HPAEpiCs) after polyhexamethylene guanidine phosphate (PHMG-p) treatment. HPAEpiCs were exposed to PHMG-p in a 10-point dose for 24 h, 48 h, and 72 h. All experiments were performed 3 times with biological replicates. Statistically significant differences were analyzed using one-way analysis of variance (ANOVA; * *p* < 0.05, ** ***p*** < 0.01, *** *p* < 0.001, **** *p *< 0.0001 versus 0 µg/mL 24 h; ^ *p* < 0.05, ^^ *p* < 0.01, ^^^ *p* < 0.001, ^^^^ *p* < 0.0001 versus 0 µg/mL 48 h; + *p* < 0.05, +  + *p* < 0.01, +  +  + *p* < 0.001, +  +  +  + *p* < 0.0001 versus 0 µg/mL 72 h.)

To determine the long-term exposure concentration that is not superficially reactive, such as cell death by PHMG-p, cell viability assays were carried out in HPAEpiCs (Fig. 1). Cytotoxicity and cell viability assays were performed at 24 h, 48 h, and 72 h. The concentration range was set to under 8 ug/ml through a preliminary experiment. The viability of HPAEpiCs decreased in a time- and dose-dependent manner following PHMG-p exposure. The survival rate was 90% with PHMG-p at a concentration of 5 to 6 µg/mL at 24 h, but the concentration gradually decreased in a time-dependent manner, and with 2 ug/mL at 72 h, the viability was approximately 90%. In contrast, a higher susceptibility to PHMG-p was reported in lung-derived IMR-90, A549, and BEAS-2B cells [19]. Taken together, HPAEpiCs constituting the internal surface area of the lung have lower susceptibility to PHMG-p treatment than the various lung-originated cells mentioned above, so it is a suitable model for identifying genetic changes that are not biased towards cell death signaling in terms of long-term PHMG-p toxicity. Considering the period for long-term sub-culture intervals with PHMG-p exposure, the maximum treatment concentration of 1 ug/ml, presenting cell viability of greater than 90% at 72 h, was selected.

### PHMG-p exposure time-dependently increased number of altered genes.

**Fig. 2:**
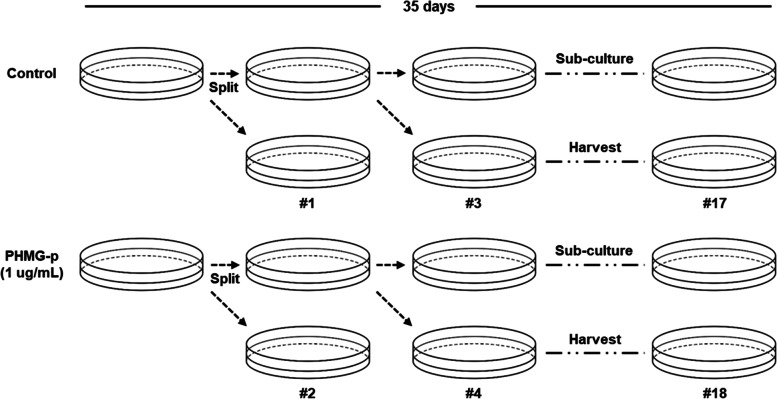
Schematic of PHMG-p low-dose exposure with long-term subculture and cell harvesting. Odd numbers indicate control samples and even numbers indicate PHMG-p-treated samples. The detailed description is present in the main text

**Fig. 3:**
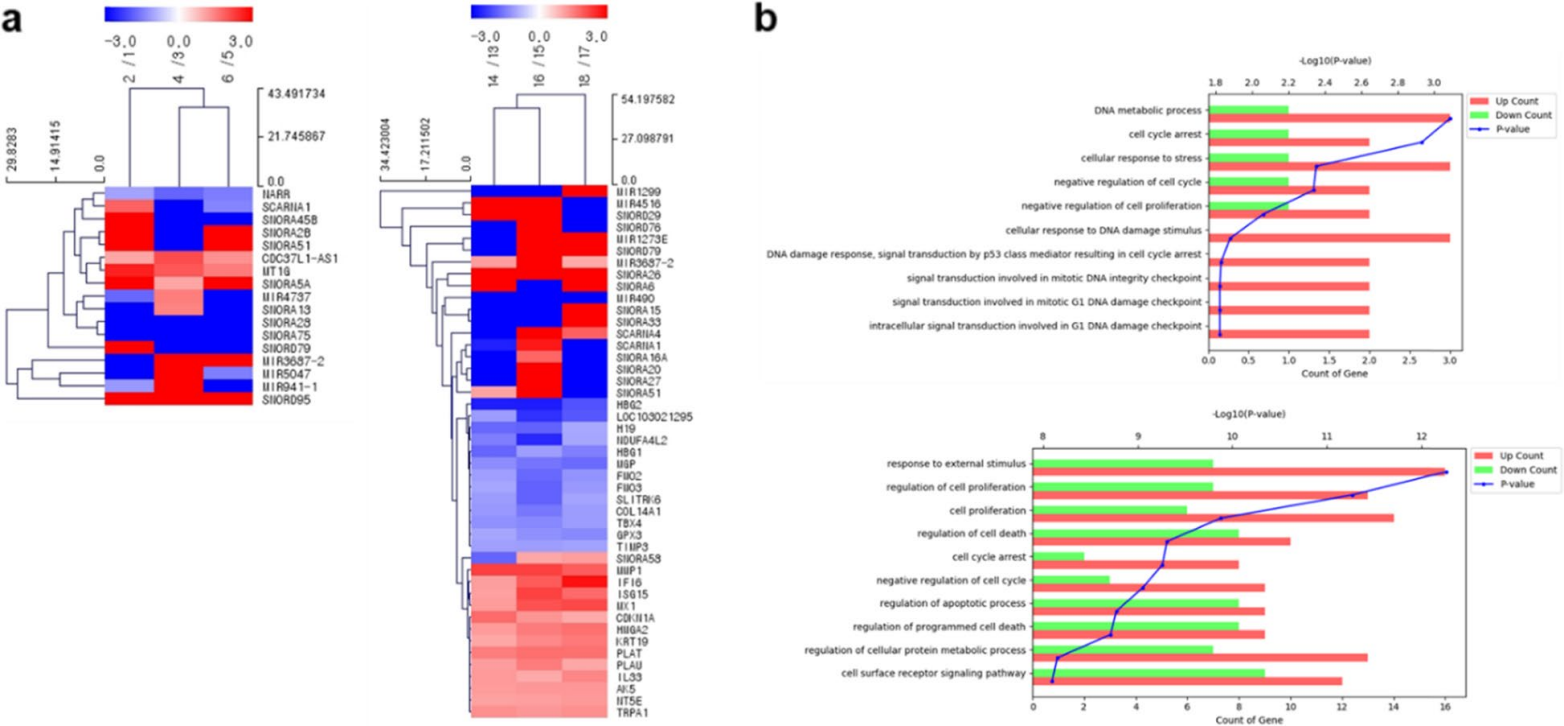
Effects of PHMG-p on the gene expression of HPAEpiCs. (a) Heatmap of selected genes (more than twofold; *P* < 0.05) in HPAEpiCs after exposure to 1 ug/mL of PHMG-p. Left panel indicates altered genes in each group of short-term treatment and right panel indicates altered genes in long-term treatment group. (b) Gene ontology enrichment analysis of the top 10 biological processes for selected genes

To investigate the genetic changes between the six sets comprising the short-term treatment group (within 10 days from 4 days; consists of 3 sets; #1 vs #2, #3 vs #4, and #5 vs #6) and the long-term treatment group (within 35 days from 27 days; consists of 3 sets; #13 vs #14, #15 vs #16, and #17 vs #18), we performed subcultures for 35 days using medium containing PHMG-p at a concentration of 1 µg/mL (Fig. 2). When heatmaps were generated with genes whose transcriptional expression changed more than twofold or less than 0.5-fold in three sets per group, only *MT1G* and *NARR* were protein-coding genes in the short-term treatment group (Table 1). Although a total of 17 genes with altered expression were selected in the three sets (1 vs #2, #3 vs #4, and #5 vs #6) constituting the short-term treatment group, genes with altered expression in the set constituting the long-term treatment group had increased to 45 genes in total. We confirmed that there were 24 protein-coding genes (*MMP1*, *IFI6*, *ISG15*, *MX1*, *CDKN1A*, *HMGA2*, *KRT19*, *PLAT*, *PLAU*, *IL-33*, *AK5*, *NT5E*, *TRPA1*, *HBG2*, *NDUFA4L2*, *HBG1*, *MGP*, *FMO2*, *FMO3*, *SLITRK6*, *COL14A1*, *TBX4*, *GPX3*, and *TIMP3*) with consistently increasing and decreasing trends in the three sets. We found that a significant number of genes were involved in multiple mechanisms related to lung cancer (Fig. 3a and Table 2). Of these genes, GO analysis indicated that in PHMG-p long-term treated HPAEpiCs, genes mainly involved in response to external stimuli and cell proliferation were upregulated. On the other hand, the genes involved in cell cycle arrest and negative regulation of the cell cycle were increased in response to short-term treatment with PHMG-p (Fig. 3b).

**Table 1:** Overview of common gene expression level changes in PHMG-p short-term treated HPAEpiCs and their reported expression changes in lung cancer

Gene symbol	Entrez ID	Description	Biotype	Fold change (FC)*			Expression	Reference(s)
				**#2/#1**	**#4/#3**	**#6/#5**		
SNORD95	619570	small nucleolar RNA, C/D box 95	snoRNA	6,000	8,540	8,720	Up	[20]
MT1G	4495	metallothionein 1G	protein coding	6.15	3.92	2.93	Up	[21, 22]
							Dn	[23]
CDC37L1-AS1	101929351	CDC37L1 antisense RNA 1 (head-to-head)	lncRNA	2.06	4.35	2.43	-	-
SNORA5A	654319	small nucleolar RNA, H/ACA box 5A	snoRNA	35.0	2.10	62.7	-	-
SNORA75	654321	small nucleolar RNA, H/ACA box 75	snoRNA	0.0328	0.0290	0.0533	Up	[20, 24]
SNORA28	677811	small nucleolar RNA, H/ACA box 28	snoRNA	0.0367	0.0169	0.0105	-	-
NARR	100861437	nine-amino acid residue-repeats	protein coding	0.479	0.289	0.349	-	-

(Dn: Down), (*: The FC value is provided as whole number, and the number of significant figures is specified as three.)

**Table 2:** Overview of common gene expression level changes in PHMG-p long-term treated HPAEpiCs and their reported expression changes in lung cancer

Gene symbol	Entrez ID	Description	Biotype	Fold change (FC)*			Expression	Reference(s)
				**#14/#13**	**#16/#15**	**#18/#17**		
SNORA26	677810	small nucleolar RNA, H/ACA box 26	snoRNA	75.6	39.7	38.6	-	-
IFI6	2537	interferon alpha inducible protein 6	protein coding	2.23	3.81	7.01	-	-
MX1	4599	MX dynamin like GTPase 1	protein coding	2.25	4.14	4.41	Up	[25]
MMP1	4312	matrix metallopeptidase 1	protein coding	4.79	4.79	3.76	Up	[26–28]
ISG15	9636	ISG15 ubiquitin-like modifier	protein coding	2.19	4.72	3.36	Up	[29]
							Dn	[30]
HMGA2	8091	high mobility group AT-hook 2	protein coding	2.27	2.95	3.20	Up	[31–34]
PLAT	5327	plasminogen activator, tissue type	protein coding	2.87	3.25	3.20	Up	[35, 36]
KRT19	3880	keratin 19	protein coding	2.04	2.64	2.97	Up	[37–39]
IL33	90865	interleukin 33	protein coding	2.30	2.01	2.68	Up	[40–42]
TRPA1	8989	transient receptor potential cation channel subfamily A member 1	protein coding	2.48	2.33	2.50	Up	[43, 44]
AK5	26289	adenylate kinase 5	protein coding	2.27	2.35	2.35	Dn	[45]
NT5E	4907	5'-nucleotidase ecto	protein coding	2.17	2.22	2.30	Up	[46–48]
PLAU	5328	plasminogen activator, urokinase (uPA)	protein coding	2.23	2.93	2.06	Up	[49–51]
MIR3687-2	103504728	microRNA 3687–2	miRNA	2.22	10,900	2.01	Up	[52, 53]
CDKN1A	1026	cyclin-dependent kinase inhibitor 1A	protein coding	3.36	2.38	2.00	Up	[54]
							Dn	[55]
TBX4	9496	T-box 4	protein coding	0.409	0.384	0.448	Dn	[56–58]
SLITRK6	84189	SLIT and NTRK like family member 6	protein coding	0.451	0.277	0.476	-	-
TIMP3	7078	TIMP metallopeptidase inhibitor 3	protein coding	0.463	0.454	0.470	Dn	[59, 60]
H19	283120	H19, imprinted maternally expressed transcript	lncRNA	0.293	0.279	0.497	Up	[61–63]
COL14A1	7373	collagen type XIV alpha 1	protein coding	0.432	0.334	0.448	Dn	[64]
FMO3	2328	flavin containing monooxygenase 3	protein coding	0.486	0.279	0.432	-	-
FMO2	2327	flavin containing monooxygenase 2	protein coding	0.457	0.295	0.415	Dn	[65, 66]
GPX3	2878	glutathione peroxidase 3	protein coding	0.486	0.409	0.387	Dn	[67, 68]
NDUFA4L2	56901	NADH dehydrogenase (ubiquinone) 1 alpha subcomplex, 4-like 2	protein coding	0.351	0.174	0.486	Up	[69]
HBG1	3047	hemoglobin subunit gamma 1	protein coding	0.291	0.454	0.358	Dn	[70, 71]
MGP	4256	matrix Gla protein	protein coding	0.395	0.328	0.299	Dn	[72]
LOC103021295	103021295	uncharacterized LOC103021295	lncRNA	0.448	0.186	0.257	-	-
HBG2	3048	hemoglobin subunit gamma 2	protein coding	0.160	0.158	0.245	Dn	[71]
MIR490	574443	microRNA 490	miRNA	0.0412	0.0163	0.0237	Dn	[73–76]

(Dn: Down), (*: The FC value is provided as whole number, and the number of significant figures is specified as three.)

**Fig. 4:**
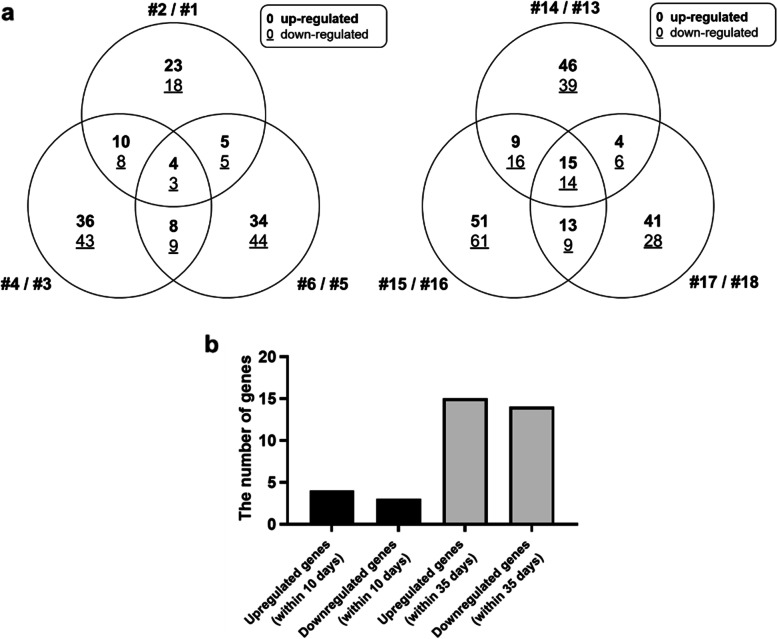
Changes in transcriptional expression levels (more than twofold or less than 0.5-fold) in PHMG-p treated HPAEpiCs. (a) Numbers of commonly changed genes are shown in a Venn diagram. Upregulated gene counts are shown in bold and downregulated gene counts are underlined. (b) Upregulated and downregulated genes at short-term treatment (within 10 days) and long-term treatment (within 35 days), respectively

From the above results, to ensure higher reliability, we selected genes with a common tendency to increase or decrease in all sets, not in each set in the same group. The numbers of changed genes are shown in a Venn diagram (Fig. 4a), and the number of genes that changed in common within each of the three sets of the short-term and long-term treatment groups are shown graphically (Fig. 4b). When the PHMG-p exposure period was prolonged, the number of overlapping genes between the two sets in each group increased from 45 to 57 genes (1.27-fold), but the number of overlapping genes between the three sets increased from 7 to 29 genes (4.14-fold) (Fig. 4a and b). These results indicated that the 29 overlapping genes in all sets were, with a high probability, altered due to long-term exposure to PHMG-p.

### PHMG-p responsive genes associated with lung cancer in PHMG-p-treated HPAEpiCs in a time-dependent manner.

We found that the expression of *SNORD95* (small nucleolar RNA, C/D box 95) was significantly elevated in PHMG-p-exposed HPAEpiC samples (Fold change (FC), #2/#1: 6,000, #4/#3: 8,540, #6/#5: 8,720). Conversely, the expression of *SNORA75* (small nucleolar RNA, H/ACA box 5A gene), *SNORA28* (small nucleolar RNA, H/ACA box 28), and *NARR* (nine-amino acid residue-repeat gene) was repressed in our results (FC, *SNORA75*, #2/#1: 0.0328, #4/#3: 0.0290, #6/#5: 0.0533; *SNORA28*, #2/#1: 0.0367, #4/#3: 0.0169, #6/#5: 0.0105; *NARR*, #2/#1: 0.479, #4/#3: 0.289, #6/#5: 0.349). *MT1G* (Metallothionein 1G) was upregulated in all three sets of short-term treatment groups (FC, #2/#1: 6.15, #4/#3: 3.92, #6/#5: 2.93) (Table 1). The expression levels of *CDC37L1-AS1* and *SNORA5A* were increased (FC, *CDC37L1-AS1*, #2/#1: 2.06, #4/#3: 4.35, #6/#5: 2.43; *SNORA5A*, #2/#1: 35.0, #4/#3: 2.10, #6/#5: 62.7); however, the transcriptional levels of these genes showed a fluctuating pattern over time (Table 1).In contrast to the results of short-term PHMG-p treatment, in the long-term treatment, the transcriptionally altered genes consisted of 24 protein-coding genes (*IFI6*, *MX1*, *MMP1*, *ISG15*, *HMGA2*, *PLAT*, *KRT19*, *IL33*, *TRPA1*, *AK5*, *NT5E*, *PLAU*, *CDKN1A*, *TBX4*, *SLITRK6*, *TIMP3*, *COL14A1*, *FMO3*, *FMO2*, *GPX3*, *NDUFA4L2*, *HBG1*, *MGP*, and *HBG2*), two lncRNAs (*H19* and *LOC103021295*), 2 microRNAs (miR-3687–2 and miR-490), and 1 snoRNA (*SNORA26*), and most of them were identified as protein-coding genes. Of these genes, *IFI6* and *MX1* tended to increase serially (FC, *IFI6*, #14/#13: 2.23, #16/#15: 3.81, #18/#17: 7.01; *MX1*, #14/#13: 2.25, #16/#15: 4.14, #18/#17: 4.41) as the exposure time of PHMG-p increased (Table 2). Upregulated levels of *MMP1*, *HMGA2*, *TRPA1* and *PLAU* (*MMP1*, #14/#13: 4.79, #16/#15: 4.79, #18/#17: 3.76; *HMGA2*, #14/#13: 2.27, #16/#15: 2.95, #18/#17: 3.20; *TRPA1*, #14/#13: 2.48, #16/#15: 2.33, #18/#17: 2.50; *PLAU*, #14/#13: 2.23, #16/#15: 2.93, #18/#17: 2.06) were identified, and the fold changes were increased by FC value. In the case of *ISG15*, *COL14A1* and *HBG2* (*ISG15*, #14/#13: 2.19, #16/#15: 4.72, #18/#17: 3.36; *COL14A1*, #14/#13: 0.432, #16/#15: 0.334, #18/#17: 0.448; *HBG2*, #14/#13: 0.160, #16/#15: 0.158, #18/#17: 0.245), they increased by FC value (Table 2). The anti-apoptotic ability of PLAT in non-small cell lung cancer (NSCLC) was increased by 2.87-, 3.25-, and 3.20-fold (FC) in sets #14/#13, #16/#15, and #18/#17, respectively. KRT19, which binds to the COOH-terminal domain of HER2 and activates HER2-Erk signaling, was upregulated in proportion to the PHMG-p exposure time (FC, #14/#13: 2.04, #16/#15: 2.64, #18/#17: 2.97). *IL33* was increased by 2.30-, 2.01-, and 2.68-fold (Table 2). The transcriptional levels of *AK5* and *NT5E* were upregulated below the FC value of 2.35-fold. *SLITRK6*, *FMO3*, and *LOC103021295*, which do not have references for their expression in lung cancer, were decreased by 0.451, 0.277, 0.476, 0.486, 0.279, 0.432, 0.448, 0.186, and 0.257 FC value grouped in 3 sets (Table 2). The expression of *GPX3* and *MGP* gradually decreased with the exposure time of PHMG-p (FC, *GPX3*, #14/#13: 0.486, #16/#15: 0.409, #18/#17: 0.387; *MGP*, #14/#13: 0.395, #16/#15: 0.328, #18/#17: 0.299). The transcriptional level of *TBX4* was decreased in all sets of the long-term treatment group (FC, #14/#13: 0.409, #16/#15: 0.384, #18/#17: 0.448). We also investigated the levels of *TIMP3*, *FMO2*, *HBG1* and *NDUFA4L2* (*TIMP3*, #14/#13: 0.463, #16/#15: 0.454, #18/#17: 0.470; *FMO2*, #14/#13: 0.457, #16/#15: 0.295, #18/#17: 0.415; *HBG1*, #14/#13: 0.291, #16/#15: 0.454, #18/#17: 0.358; *NDUFA4L2*, #14/#13: 0.351, #16/#15: 0.174, #18/#17: 0.486), and the fold changes were decreased by FC value (Table 2).It was confirmed that the altered fold change of non-coding RNAs (ncRNAs) is large scale (the absolute value of FC, from 2.01 to 10,900) compared to the degree of change in the protein-coding genes (the absolute value of FC, from 2.01 to 7.01) in the long-term treatment group. The genes whose expression was altered were *SNORA26*, *MIR3687-2* and *MIR490* (*SNORA26*, #14/#13: 75.6, #16/#15: 39.7, #18/#17: 38.6; *MIR3687-2*, #14/#13: 2.22, #16/#15: 10,900, #18/#17: 2.01; *MIR490*, #14/#13: 0.0412, #16/#15: 0.0163, #18/#17: 0.0237), all of which consist of ncRNAs. *MIR3687-2* and *MIR490* increased in all sets, but in the case of MIR3687-2, the FC value increased significantly (FC = 10,900), especially in sets #16 and #15. In the case of *MIR490*, it was confirmed that it decreased significantly in all sets (Table 2).

### RT-qPCR validation of altered protein coding genes

**Fig. 5:**
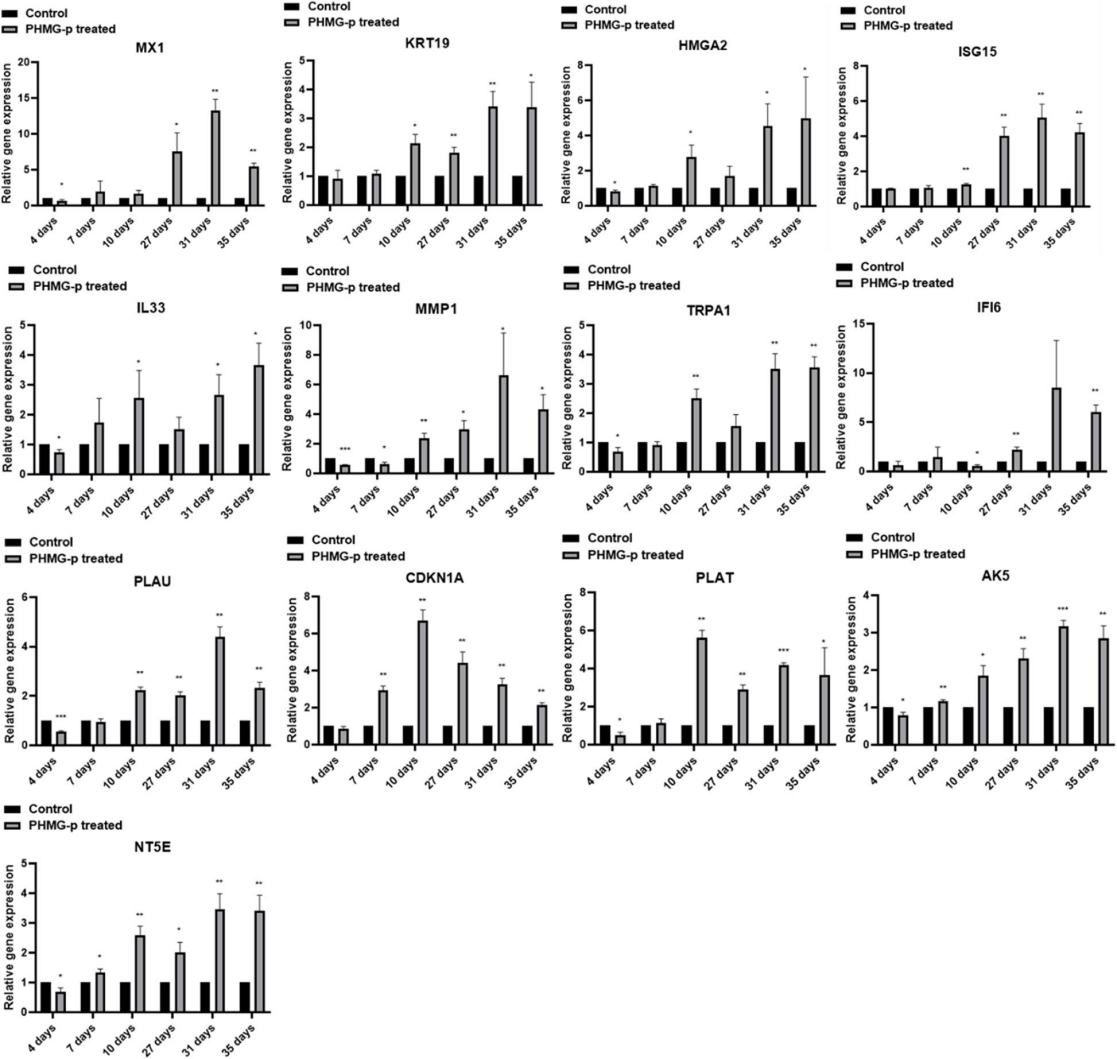
RT-qPCR validation for upregulated protein coding genes in the long-term treatment group. To validate the upregulated candidate genes on total RNA sequencing, the expression of *MX1*, *KRT19*, *HMGA2*, *ISG15*, *IL33*, *MMP1*, *TRPA1*, *IFI6*, *PLAU*, *CDKN1A*, *PLAT*, *AK5* and *NT5E* was determined by RT-qPCR. All selected genes were verified to be increased in the long-term treatment group (within 35 days from 27 days). All experiments were performed 3 times with technical replicates

**Fig. 6:**
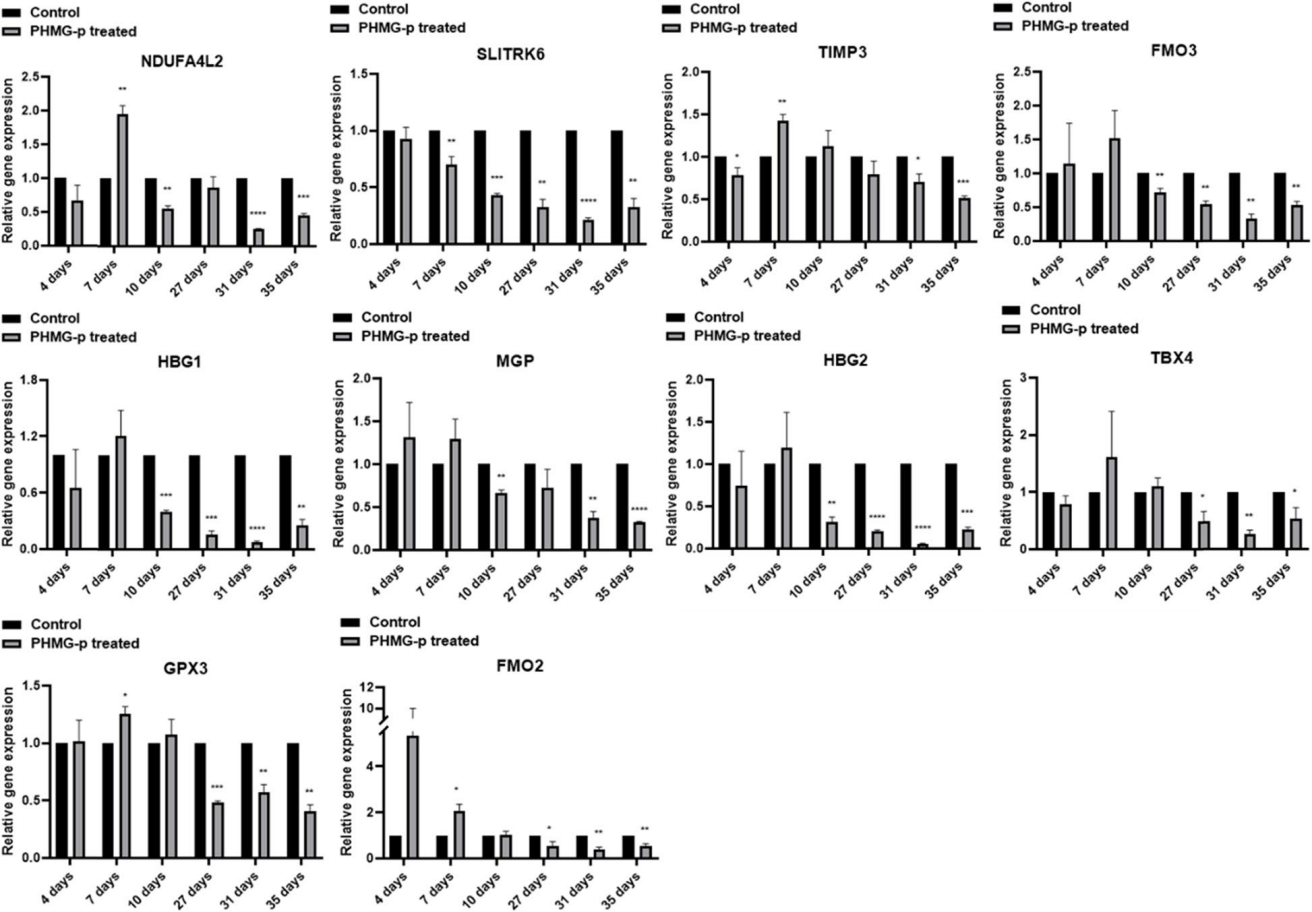
RT-qPCR validation for downregulated protein coding genes in the long-term treatment group. To validate the upregulated candidate genes on total RNA sequencing, the expression of *NDUFA4L2*, *SLITRK6*, *TIMP3*, *FMO3*, *HBG1*, *MGP*, *HBG2*, *TBX4*, *GPX3*, and *FMO2* was determined by RT-qPCR. All selected genes were verified to be decreased in the long-term treatment group. All experiments were performed 3 times with technical replicates

To validate the candidate genes selected according to the criteria (more than 2.0-fold or less than 0.5-fold) on total RNA sequencing, RT-qPCR was performed. To confirm the increased transcriptional level in upregulated candidates, the expression of *MX1*, *KRT19*, *HMGA2*, *ISG15*, *IL33*, *MMP1*, *TRPA1*, *IFI6*, *PLAU*, *CDKN1A*, *PLAT*, *AK5*, and *NT5E* was determined by RT-qPCR. In particular, it was confirmed that *MX1* significantly increased from 5.4-fold to less than 13.3-fold in the long-term treated sets (Fig. 5). Based on the total RNA sequencing results, *CDKN1A* was not selected because it did not meet the criteria in the PHMG-p short-term treatment group (set of 4 days), but in this validation phase, it increased up to 6.7-fold in the short-term group (set of 10 days). As a result, it was confirmed that *CDKN1A* increased 3.5-fold in the short-term treatment group and 3.3-fold in the long-term treatment group (Fig. 5). All selected protein-coding genes were found to be increased in the long-term treatment group (within 35 days from 27 days). Unlike other candidate genes, it has been reported that *AK5* expression was reduced due to methylation of the promoter region within the CpG islands in lung adenocarcinoma [45]. The downregulated expression of *NDUFA4L2*, *COL14A1*, *SLITRK6*, *TIMP3*, *FMO3*, *HBG1*, *MGP*, *HBG2*, *TBX4*, *GPX3*, and *FMO2* was also confirmed (Fig. 6). However, the basal expression of *COL14A1* was very low, and the cycle threshold (Ct) value of RT-qPCR was not measurable; therefore, it was excluded from Fig. 6. In addition, contrary to the downregulated tendency, *NDUFA4L2* was overexpressed in human NSCLC, reported to occur under hypoxic conditions, one of the characteristics of cancer. Its overexpression is a key factor for maintaining NSCLC growth [69]. The tendency of transcriptional alteration of all candidate genes in RT-qPCR was 100% consistent with the total RNA sequencing results because the candidate genes were selected based on matching in all three sets (#13 vs #14, #15 vs #16, and #17 vs #18).

### Clinical significance of *ISG15, MMP1, TRPA1, KRT19, FMO3, COL14A1, FMO2* and *TIMP3* in patients with lung adenocarcinoma

**Fig. 7:**
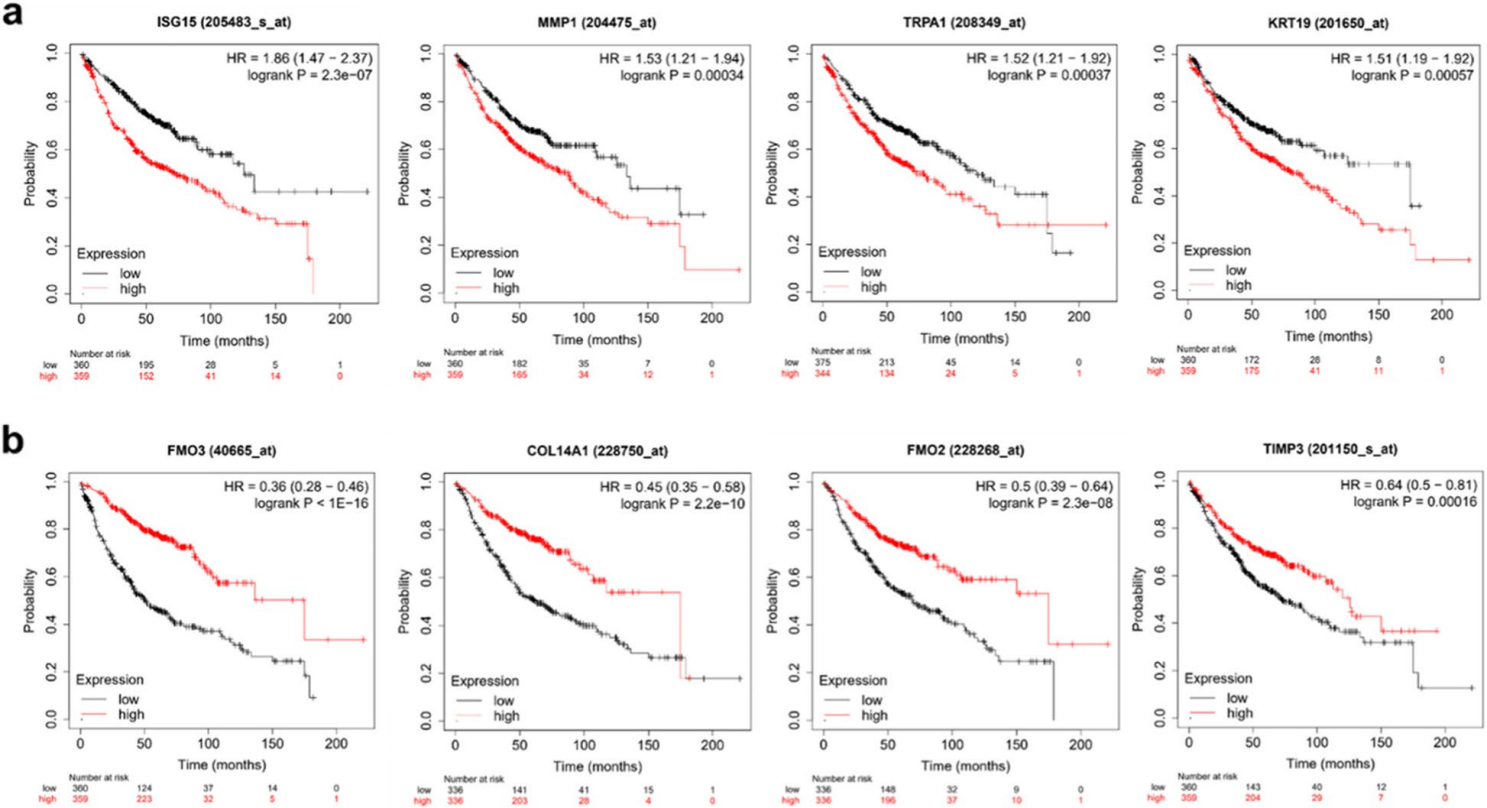
Clinical significance of selected genes in patients with lung adenocarcinoma. Kaplan–Meier survival analysis generated for groups of patients based on the expression levels of upregulated genes (*ISG15*, *MMP1, TRPA1* and *KRT19*) (a) and downregulated genes (*FMO3, COL14A1*, *FMO2* and *TIMP3*) (b) in the database

To estimate the survival function of *ISG15, MMP1, TRPA1, KRT19,* and *PLAU*, we performed KM plotter analysis generated for groups of lung adenocarcinoma patients based on their expression levels. As indicated in Fig. 7a and S1a, lung adenocarcinoma patients with high expression levels of *ISG15*, *MMP1*, *TRPA1, KRT19,* and *PLAU* (red line) were significantly associated with poor survival rates (log rank P value: 2.3e-07, 0.00034, 0.00037, 0.00057, and 0.023, respectively) as compared to those with low expression (black line). In addition to the results of increased gene expression, downregulated *FMO3*, *COL14A1*, *FMO2*, *TIMP3*, and *SLITRK6* also showed a poor survival rate (log rank P value: < 1.0e-16, 2.2e-10, 2.3e-0.8, 0.00016, and 0.0042, respectively) compared to their high expression (Figs. 7b and S1b Figure). Hazard ratio (HR) scores of the five upregulated genes were 1.86 (1.47 · 2.37), 1.53 (1.21 · 1.94), 1.52 (1.21 · 1.92), 1.51 (1.19 · 1.92), and 1.31 (1.04 · 1.65), respectively. Also, HR scores of five downregulated genes were 0.36 (0.28 to 0.46), 0.45 (0.35 to 0.58), 0.50 (0.39 to 0.64), 0.64 (0.50 to 0.81), and 0.70 (0.55 to 0.90). These data indicate that upregulated transcriptional levels of *ISG15*, *MMP1*, *TRPA1*, *KRT19*, and *PLAU* and decreased expression of *FMO3*, *COL14A1*, *FMO2*, *TIMP3*, and *SLITRK6* are highly associated with unfavorable overall survival of lung adenocarcinoma patients.

## Discussion

In this study, we confirmed the distorted homeostasis of oncogenes and tumor suppressor genes that change in a time-dependent manner by PHMG-p in normal human lung alveolar cells. Previous in vitro studies have investigated the effect of PHMG-p exposure on lung cells and (i) focused on fibrotic inflammation, (ii) used immortalized cells or lung cells derived from malignant tumors, and (iii) identified a molecular mechanism that changes with short-term PHMG-p treatment [2, 9]. To overcome these limitations, we (i) focused on carcinogenesis-related genetic changes, (ii) introduced human type I and II normal alveolar cells that make up most of the inner surface of the lungs, and (iii) obtained results through a long-term exposure procedure. In the case of RNA sequencing, instead of repeating the experiment with same conditions, 3 groups were selected from short-term treatment and 3 groups from long-term treatment to increase the reliability of this research. Moreover, each gene in the Venn diagram of Fig. 4 was selected by reflecting the fold change, p-value, and normalized data (log2) values rather than reflecting only a single variable. Therefore, it is likely that the differences in the selected genes between each group will be increase. However, we think that the number of genes that change in common during long-term culture is more reliable because they were commonly selected despite applying 3 variables mentioned above. In addition, we suggested the possibility that PHMG-p can increase lung carcinogenesis by performing GO, heatmap cluster, differentially expressed gene (DEG) analysis including pre-mRNA, lncRNA, and miRNA, and KM plotter analysis with selected genes.

Within 10 days of exposure to PHMG-p, two protein-coding genes and five non-coding genes were altered. In the PHMG-p group treated for 27–35 days, 24 protein-coding and 5 non-coding genes were identified in the PHMG-p group. Interestingly, in our selection criteria for candidate genes, the number of ncRNAs did not change, but the number of protein-coding genes increased by a factor of 12. In addition, it was found that the degree of change in the case of ncRNAs was largely compared to the degree of change in the protein-coding genes (Tables 1 and 2), but it is not clear whether this phenomenon is a transcriptional characteristic of ncRNA itself or a characteristic of reactivity in PHMG-p treatment.

To confirm whether the selected genes are related to lung cancer, we searched all published papers and also listed genes that contradict our hypothesis to eliminate bias. *MT1G* was upregulated in all three short-term treatment groups. Although the expression of *MT1G* in breast, thyroid cancer, and hepatocellular carcinoma was downregulated compared to that in non-cancerous tissue, it has been reported that the expression of *MT1G* in NSCLC is higher than that in non-malignant lung tissues [21, 77–79]. Furthermore, *MT1G* was enriched in the most aggressive large-cell lung carcinoma, and high expression of *MT1G* correlated with poor prognostic values ​​in 24 lung large-cell lung carcinomas [22]. However, only one group contended that the expression of *MT1G* was lower in lung cancer tissues than in peri-cancer tissues [23]. In a different trend from altered genes, *CDKN1A* mRNA gradually increased in the short-term treatment group and continuously decreased in the long-term treatment group. Inferring from the above results, the possibility exists that expression of *CDKN1A* was upregulated to inhibit cell growth in the short term, but it is thought that as many oncogenes increase, they lose their ability to regulate homeostasis with respect to growth. IL-33, a member of the IL-1 family that promotes the production of Th2-related cytokines, was increased in our study (Table 2). In addition, reports on the elevated expression levels of *IL33* and *NT5E* have focused on NSCLC [40–42, 46–48]. Indeed, blockage of IL-33 is known to prevent the growth of NSCLC by inhibiting M2 macrophage polarization and reducing the accumulation of Treg cells in the tumor microenvironment [40]. NT5E is a novel target for the treatment of many cancer types, and various NT5E/CD73 inhibitors are currently being tested in clinical trials. In NSCLC, the level of *NT5E*, a target gene of miR-30a-5p, is increased due to decreased expression of miR-30a-5p. *NT5E* also contributes to the survival of NSCLC by inhibiting its function by trapping miR-134 [46, 48]. *IFI6* and *MX1* tended to increase serially as the exposure time of PHMG-p increased. The IFI6 protein plays a central role in resistance to apoptosis in various cancer types [80, 81], and MX1 protein levels are increased in lung adenocarcinoma [25]. Furthermore, it has been reported that *MMP1, HMGA2, TRPA1*, and *PLAU* are highly expressed in various subtypes of lung cancer [26–28, 31–34, 43, 44, 49–51]. However, *ISG15*, *COL14A1* and *HBG2* are mainly decreased in lung adenocarcinoma [30, 64, 70, 71].

TBX4, which is involved in the regulation of embryonic developmental processes, is downregulated in lung cancer [56–58], and it was decreased in all sets of the long-term treatment group. TIMP3 is also known to play a role in inducing apoptosis and suppressing NSCLC growth [59, 60]. The *COL14A1* promoter region was confirmed to be hypermethylated, with a probability of 60.4% in 48 NSCLC patient samples [64]. Although the role of FMO2, whose main function is an NADPH-dependent enzyme, in tumorigenesis is still unclear, it has been reported to play a role as a tumor suppressor in lung adenocarcinoma [65, 66]. GPX3, a scavenger of reactive oxygen species, is known to inhibit the growth, invasion, and migration of various lung cancer cells, including h157, h460, h1299, h1650 h1975, and A549 [67, 68]. *HBG1* and *HBG2*, the gamma globin genes, have been reported to have low expression in NSCLC, including adenocarcinoma and squamous cell carcinoma [70, 71]. MGP expression was found to be reduced during the symptomatic illness stage in lung cancer [72].

In malignant tumors, there are reports of distorted homeostasis between oncogenes and tumor suppressor genes, including failure to regulate the expression levels of ncRNAs [82, 83]. Most of these ncRNAs cannot directly bind to ribosomes and can be translated into proteins, but they can interact with other coding genes or non-coding genes; by binding to several proteins, they affect the interactions between proteins and serve as sponges for microRNAs [84, 85]. The expression of *SNORD95* was upregulated in PHMG-p-exposed samples, and it was also reported that *SNORD95* was increased in 11 lung squamous cell carcinoma and 11 lung adenocarcinoma samples compared to their matched normal samples [86]. Conversely, the expression of *SNORA75*, *SNORA28*, and *NARR* was downregulated in our study. However, there are no previous reports that the three genes described above are decreased in lung cancer. In the case of *MIR3687-2*, the FC value was significantly increased, especially in the #16/#15 set. In the case of *MIR490*, a significant decrease was observed in all sets. Therefore, there is a need to find the putative target genes of microRNA-3687 and microRNA-490-3p and -5p. *FGFRL1* (fibroblast growth factor receptor-like 1), *NCS1* (neuronal calcium sensor 1), *UCN2* (urocortin 2), *TMEM167B* (transmembrane protein 167B), and *CDR1* (cerebellar degeneration-related protein 1) are predicted targets of miR-3687. *VDAC1* (voltage-dependent anion channel 1), *TMOD3* (tropomodulin 3), *COMMD10* (COMM domain containing 10), *HNRNPA1* (heterogeneous nuclear ribonucleoprotein A1), *PIP4K2B* (phosphatidylinositol-5-phosphate 4-kinase, type II, beta) are putative targets of miR-490-3p. *ZNF627* (zinc finger protein 627), *TTC29* (tetratricopeptide repeat domain 29), *BTC* (betacellulin), *CST8* (cystatin 8), and *LILRA3* (leukocyte immunoglobulin-like receptor, subfamily A (without TM domain), member 3) are putative targets of miR-490-5p which were predicted in the microRNA target prediction programs, such as TargetScan and miRDB. Surprisingly, it was recently reported that among the two miRNAs that change in a PHMG-p-dependent manner, miR-3687 is increased in lung squamous cell carcinoma and miR-490 is decreased in lung squamous cell carcinoma and lung adenocarcinoma [52].

Furthermore, since the HPAEpiCs used in this research are known to consist of pulmonary alveolar type I (AT1) and alveolar type II (AT2), we identified AT1 and AT2 specific gene markers. AT1-specific genes, IGFBP2, CAV1, and CAV2, indicated a tendency to increase as the subculture days prolonged. Therefore, we inferred that AT2 cells in HPAEpiCs gradually differentiated into AT1 cells. In addition, considering the normalized data, we confirmed that most HPAEpiCs consisted of AT1 cells, and that AT2 cells were also included (Table S2) [87–92].

Our follow-up studies will be focused on delineating the transcriptional regulation of ncRNAs including microRNA, snoRNA, and lncRNA altered by PHMG-p, discovering their interacting genes. Also, there is a need to investigate the molecular mechanisms in human bronchial and tracheal epithelial cells that are not limited to alveolar epithelial cells but are primarily exposed upon inhalation of respirable particles containing PHMG-p.

Taken together, for the first time, we confirmed and validated the distorted regulation of repetitively altered genes in three experimental sets of PHMG-p long-term treatment groups using normal pulmonary alveolar cells, which constitute the majority of the internal surface of ​​the lung. In addition, most of the altered genes are closely related to lung cancer and the survival rate of patients with lung adenocarcinoma.

**Conclusions.**

Based on our description, we suggest that PHMG-p, as the main gradient of humidifier disinfectants, has a carcinogenic potential in normal human lung alveolar cells in case of long-term exposure.

## Supplementary Information

**Additional file 1. Supplementary Figure 1. **Clinical significance of selected genes in patient with lung adenocarcinoma.

**Additional file 2. Supplementary Table 1.** Primers sequence

**Additional file 3. Supplementary table 2. **Evaluation of expression of AT1 and AT2 cell specific genes

## Data Availability

All data generated or analyzed during this study are included in this published article.

## Abbreviations

PHMG-p: Polyhexamethylene guanidine phosphate
HDs: Humidifier disinfectants
HDLI: HD-associated lung injury
HPAEpiCs: Human pulmonary alveolar epithelial cells
CT: Computed tomography
GGO: Ground glass opacity
PHMB: Polyhexamethylene biguanide
EMT: Epithelial-to-mesenchymal transition
LncRNA: Long non-coding RNA
NcRNA: Non-coding RNA
