# Supplementary material for: Analysis of lung cancer-related genetic changes in long-term and low-dose polyhexamethylene guanidine phosphate (PHMG-p) treated human pulmonary alveolar epithelial cells
Source: BMC Pharmacol Toxicol. 2022 Mar 30;23:19. doi: 10.1186/s40360-022-00559-5 (PMC8969249; doi:10.1186/s40360-022-00559-5)
Supplement: Supplementary file 1 — Additional file 1. Supplementary Figure 1. Clinical significance of selected genes in patient with lung adenocarcinoma. [file 40360_2022_559_MOESM1_ESM.docx]

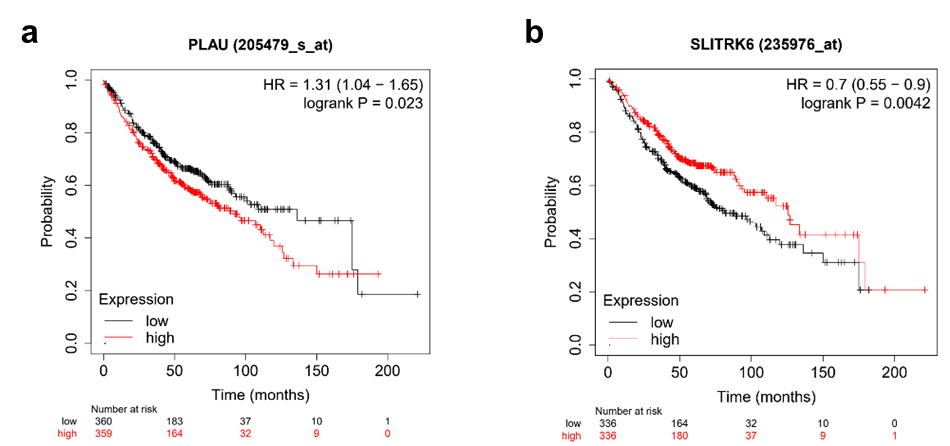


Supplementary Fig. 1 Clinical significance of selected genes in patients with lung adenocarcinoma. Kaplan-Meier survival analysis generated for groups of patients based on the expression levels of up-regulated *PLAU* (A) and down-regulated *SLITRK6* (B) in the database include GEO, EGA and TCGA.
