# Supplementary material for: Analysis of lung cancer-related genetic changes in long-term and low-dose polyhexamethylene guanidine phosphate (PHMG-p) treated human pulmonary alveolar epithelial cells
Source: BMC Pharmacol Toxicol. 2022 Mar 30;23:19. doi: 10.1186/s40360-022-00559-5 (PMC8969249; doi:10.1186/s40360-022-00559-5)
Supplement: Supplementary file 2 — Additional file 2. Supplementary Table 1. Primers sequence [file 40360_2022_559_MOESM2_ESM.docx]

**Supplementary Table 1** Primers sequence

| Gene name | Sequence (5’ → 3’) | Gene name | Sequence (5’ → 3’) |
| --- | --- | --- | --- |
| *NDUFA4L2* | Forward: CTGGGACAGAAAGAACAACCCG  Reverse: CAGCCTGGCTTAGAAGTCTGGC | ***IFI6*** | Forward: TGATGAGCTGGTCTGCGATCCT  Reverse: GTAGCCCATCAGGGCACCAATA |
| *SLITRK6* | Forward: CAACTTCCAGGACCTTACTGCC  Reverse: GGATTTTGCGGAGGAGGTCTCA | ***MX1*** | Forward: GGCTGTTTACCAGACTCCGACA  Reverse: CACAAAGCCTGGCAGCTCTCTA |
| *TIMP3* | Forward: TACCGAGGCTTCACCAAGATGC  Reverse: CATCTTGCCATCATAGACGCGAC | ***MMP1*** | Forward: ATGAAGCAGCCCAGATGTGGAG  Reverse: TGGTCCACATCTGCTCTTGGCA |
| *COL14A1* | Forward: CACAAACCTCCTCAGCGGAATG  Reverse: GGCTTGGAGATTGGTAACACCC | ***ISG15*** | Forward: CTCTGAGCATCCTGGTGAGGAA  Reverse: AAGGTCAGCCAGAACAGGTCGT |
| *TBX4* | Forward: CTCAAGCCTCTTCTATCACTGCC  Reverse: GGGAACGGAAATAGTGATCCTCC | ***HMGA2*** | Forward: GAAGCCACTGGAGAAAAACGGC  Reverse: GGCAGACTCTTGTGAGGATGTC |
| *FMO3* | Forward: TGGAAAGCGTGTCCTGGTGGTT  Reverse: TCATCACCCAGGAGCCACTTCT | ***PLAT*** | Forward: TGGTGCTACGTCTTTAAGGCGG  Reverse: GCTGACCCATTCCCAAAGTAGC |
| *FMO2* | Forward: GGAAAACGCATCCTGGTGATTGG  Reverse: GCCATCTTCAGAGATACGGCTC | ***KRT19*** | Forward: AGCTAGAGGTGAAGATCCGCGA  Reverse: GCAGGACAATCCTGGAGTTCTC |
| *GPX3* | Forward: TACGGAGCCCTCACCATTGATG  Reverse: CAGACCGAATGGTGCAAGCTCT | ***IL-33*** | Forward: GCCTGTCAACAGCAGTCTACTG  Reverse: TGTGCTTAGAGAAGCAAGATACTC |
| *HBG1* | Forward: GGAAGATGCTGGAGGAGAAACC  Reverse: GTCAGCACCTTCTTGCCATGTG | ***TRPA1*** | Forward: TCCTCTCCATCTGGCAGCAAAG  Reverse: GGACGCATGATGCAAAGCTGTC |
| *MGP* | Forward: CCTCAGCAGAGATGGAGAGCTA  Reverse: ATGGCGTAGCGTTCGCAAAGTC | ***AK5*** | Forward: TGATTGACGGCTATCCTCGGGA  Reverse: TTGGAGAAGGCGGTTGGTCATG |
| *HBG2* | Forward: GGAAGATGCTGGAGGAGAAACC  Reverse: GTCAGCACCTTCTTGCCATGTG | ***NT5E*** | Forward: AGTCCACTGGAGAGTTCCTGCA  Reverse: TGAGAGGGTCATAACTGGGCAC |
| *CDKN1A* | Forward: AGGTGGACCTGGAGACTCTCAG  Reverse: TCCTCTTGGAGAAGATCAGCCG | ***PLAU*** | Forward: GGCTTAACTCCAACACGCAAGG  Reverse: CCTCCTTGGAACGGATCTTCAG |
| *GAPDH* | Forward: GTCTCCTCTGACTTCAACAGCG  Reverse: ACCACCCTGTTGCTGTAGCCAA |  |  |
