# Supplementary material for: Analysis of lung cancer-related genetic changes in long-term and low-dose polyhexamethylene guanidine phosphate (PHMG-p) treated human pulmonary alveolar epithelial cells
Source: BMC Pharmacol Toxicol. 2022 Mar 30;23:19. doi: 10.1186/s40360-022-00559-5 (PMC8969249; doi:10.1186/s40360-022-00559-5)
Supplement: Supplementary file 3 — Additional file 3. Supplementary table 2. Evaluation of expression of AT1 and AT2 cell specific genes [file 40360_2022_559_MOESM3_ESM.docx]

**Supplementary Table 2** Evaluation of expression of AT1 and AT2 cell-specific gene

|  |  | **Normalized data of RNA-sequencing raw data** | | | | | |
| --- | --- | --- | --- | --- | --- | --- | --- |
|  |  | **Short-term group** | | | **Long-term group** | | |
|  | **Genes** | **#1** | **#3** | **#5** | **#13** | **#15** | **#17** |
| **AT1 specific genes** | **IGFBP2** | 16.51 | 23.82 | 49.32 | 120.8 | 113.6 | 167.3 |
|  | **CAV1** | 137.2 | 123.1 | 199.6 | 300.5 | 391.5 | 476.4 |
|  | **CAV2** | 27.59 | 33.92 | 55.68 | 83.87 | 95.74 | 105.3 |
| **AT2 specific genes** | **SFTPA1** | 1.025 | 1.027 | 1.030 | 1.056 | 1.000 | 1.000 |
|  | **SFTPC** | 1.000 | 1.000 | 1.000 | 1.000 | 1.000 | 1.000 |
|  | **SFTPD** | 1.044 | 1.048 | 1.261 | 1.000 | 1.000 | 1.137 |
